# Random forest-integrated analysis in AD and LATE brain transcriptome-wide data to identify disease-specific gene expression

**DOI:** 10.1371/journal.pone.0256648

**Published:** 2021-09-07

**Authors:** Xinxing Wu, Chong Peng, Peter T. Nelson, Qiang Cheng

**Affiliations:** 1 University of Kentucky, Lexington, Kentucky, United States of America; 2 Qingdao University, Qingdao, Shandong, China; Nathan S Kline Institute, UNITED STATES

## Abstract

Alzheimer’s disease (AD) is a complex neurodegenerative disorder that affects thinking, memory, and behavior. Limbic-predominant age-related TDP-43 encephalopathy (LATE) is a recently identified common neurodegenerative disease that mimics the clinical symptoms of AD. The development of drugs to prevent or treat these neurodegenerative diseases has been slow, partly because the genes associated with these diseases are incompletely understood. A notable hindrance from data analysis perspective is that, usually, the clinical samples for patients and controls are highly imbalanced, thus rendering it challenging to apply most existing machine learning algorithms to directly analyze such datasets. Meeting this data analysis challenge is critical, as more specific disease-associated gene identification may enable new insights into underlying disease-driving mechanisms and help find biomarkers and, in turn, improve prospects for effective treatment strategies. In order to detect disease-associated genes based on imbalanced transcriptome-wide data, we proposed an integrated multiple random forests (IMRF) algorithm. IMRF is effective in differentiating putative genes associated with subjects having LATE and/or AD from controls based on transcriptome-wide data, thereby enabling effective discrimination between these samples. Various forms of validations, such as cross-domain verification of our method over other datasets, improved and competitive classification performance by using identified genes, effectiveness of testing data with a classifier that is completely independent from decision trees and random forests, and relationships with prior AD and LATE studies on the genes linked to neurodegeneration, all testify to the effectiveness of IMRF in identifying genes with altered expression in LATE and/or AD. We conclude that IMRF, as an effective feature selection algorithm for imbalanced data, is promising to facilitate the development of new gene biomarkers as well as targets for effective strategies of disease prevention and treatment.

## Introduction

Dementia represents a set of slowly progressing neurodegenerative disorders with enormous public health impact, caused by a number of different underlying diseases. Alzheimer’s disease (AD) is one of the most common contributors to the neurocognitive disorder syndrome. Neuropathologically, AD is characterized by the accumulation of amyloid plaques and neurofibrillary tangles (NFTs). Currently, there is no treatment or effective preventative strategy. Further, a clear understanding of the causes of AD remains elusive.

Recently, limbic-predominant age-related TDP-43 encephalopathy (LATE) was defined [1]. LATE is a TDP-43 proteinopathy and generally affects persons aged 80 years and above. Clinically, LATE mimics AD-type dementia syndrome; LATE may be presented in isolation, or it could be comorbid with AD [2]. Therefore, it is often difficult to distinguish LATE from AD. In addition, existing research has revealed that AD, as a chronic age-related neurodegenerative disease, usually starts slowly and the cognitive deterioration of LATE is even slower than AD individually; however, AD-LATE comorbid disease typically causes a more rapid clinical decline than either of them individually. There are no effective techniques to confidently diagnose LATE or distinguish LATE from AD with clinically available biomarkers, including disease-associated genes. More detailed clinical differences and associations between AD and LATE are summarized in Table 1.

**Table 1:** LATE vs. AD.

	LATE	AD
**Discovery**	Nelson et al., 2019	Alzheimer, 1906
**Age**	Usually 80+	Usually 65+
**Clinical features**	LATE is slower than AD, but AD plus LATE will cause a more rapid decline	
**Correlation**	About a quarter of AD patients actually have LATE, which mimics AD in syndrome	
**Pathologic biomaker**	TDP-43	A*β* and tau
**Measurement**	TDP-43	Braak and CERAD

Another type of dementia, frontotemporal dementia (FTD) (Also known as Pick’s disease after Arnold Pick, who first noticed a patient with distinct symptoms affecting language in 1892), is also related to the tau and TDP-43 proteins; however, LATE usually can be distinguished from FTD, because FTD typically affects people under age 60 while LATE affects older people, and LATE neuropathologic change has a relatively restricted neuroanatomical distribution of TDP-43 proteinopathy [3].

In the present study, we focused on the differentiation of LATE, AD, and comorbid AD+LATE using transcriptome-wide data, and the identification of putative disease-related genes. Typically, the clinical samples for patients and controls are highly imbalanced (i.e., the number of controls is generally manyfold larger than that of patients), thus rendering it challenging to apply most existing machine learning algorithms directly to analyze such datasets to find differentiating features. To meet this challenge, we develop a novel, integrated algorithm, IMRF, to identify the disease-related genes by classifying AD+LATE comorbid, pure LATE, pure AD, and control subjects in imbalanced transcriptome-wide data. IMRF systematically integrates multiple RFs, it can effectively exploit differentiating features implied in imbalanced data. IMRF, as a feature selection algorithm, empirically achieves better performance than several potential RF-base algorithms, including RF with class weighting (abbreviated as RF-CW), RF with bootstrap class weighting (abbreviated as RF-BCW), and RF with random undersampling (abbreviated as RF-U) [4, 5], and existing feature selection algorithms, including feature selection using stochastic gates (abbreviated as STG) [6], least absolute shrinkage and selection operator (abbreviated as Lasso) [7], univariate feature selection (abbreviated as UFS) https://scikit-learn.org/stable/modules/feature_selection.html; see Figs 8 and 10.

Here we employed three existing clinical or neuropathological diagnostic criteria to categorize whether a subject has AD and/or LATE: 1) Braak score [8, 9], which is an ordinal measure to delineate the distribution and severity of NFT pathology with seven stages 0–6; 2) CERAD score [9, 10], which is a semiquantitative measure with four grades 1–4 to describe the neuritic plaque density; 3) TDP-43 stage [11], which has four grades to measure the TDP-43 distribution, or, a recommended dichotomy version with values 0 and 1 by the Rush AD Center (RADC). We used the first two scores for recognizing subjects with AD and the third criterion for LATE.

## Materials and methods

RF [12] is an ensemble learning algorithm that has been widely used. For classification and feature selection, RF is typically suitable for balanced data, and usually has degraded performance on highly imbalanced data. To address this issue, different variants of RF have been proposed, including RF-CW, RF-BCW, and RF-U [4, 5]. RF-CW assigns different costs to misclassifications in different classes and achieves a balance between precision and recall through cost-sensitive learning. In this process, the feature importances would be also rescaled by the cost weights. Since the class weights may have a wide range, the ranked features using the feature importances appear less reliable. Also, it may artificially change the distribution of training samples. RF-U extends RF by performing undersampling of the majority classes with replacement for each iteration of RF, thus making the samples used for training different for each iteration and the testing performance less stable. RF-BCW constructs each decision tree by bootstrapping samples, and it may lead to less effective ensemble learning and feature selection because the distribution of samples for each tree is different.

**Fig 1:**
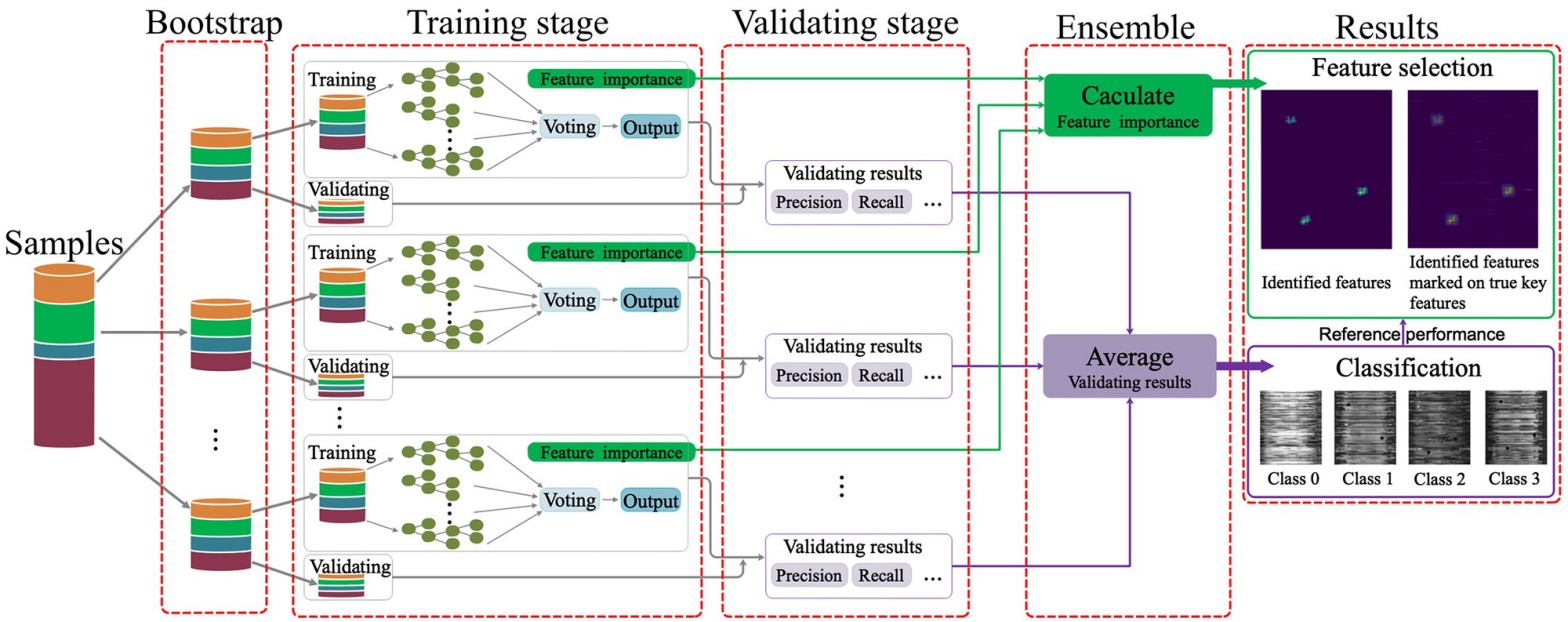
Overall scheme of IMRF. As an illustration, we show the use of IMRF on synthetic dataset with or without tiny black points for visualization.

In this study, our main purpose is to identify the disease-related genes for LATE, AD, and LATE+AD based on imbalanced transcriptome-wide data. The existing variants of RF mainly focus on how to classify imbalanced data rather than how to select a subset of important features from such data. If only a part of the samples from the majority class(es) were used, it would under-utilize the clinically valuable data and obtain misleading feature importance. Also, if the majority class(es) are boostrapped many times while the remaining minority class(es) are kept the same, then the subset of samples used in constructing the decision trees by RF would be almost the same for different bootstrapping iterations, leading to degraded ensemble learning performance. To remedy the above drawbacks, we develop a novel RF-based approach, IMRF, by integrating multiple RFs to sufficiently analyze and discriminate the imbalanced samples in different classes. For class-imbalanced data, it can not only effectively achieve a more balanced precision-recall tradeoff, but also aggregate the feature importances calculated from multiple RFs to identify the informative features. The overall scheme of IMRF is shown in Fig 1. It consists of five stages: Firstly, bootstrapping to balance the samples, which splits all the samples into several balanced subsets; Secondly, training each subset of samples with balanced classes by RF to evaluate learning parameters such as feature importance; Thirdly, computing the classification results, such as precision, recall, F1 score, and accuracy, for multiple RFs on validation samples; Fourthly, averaging the feature importances and validating results from different RFs; Finally, obtaining the classification and feature identification results.

**Fig 2:**
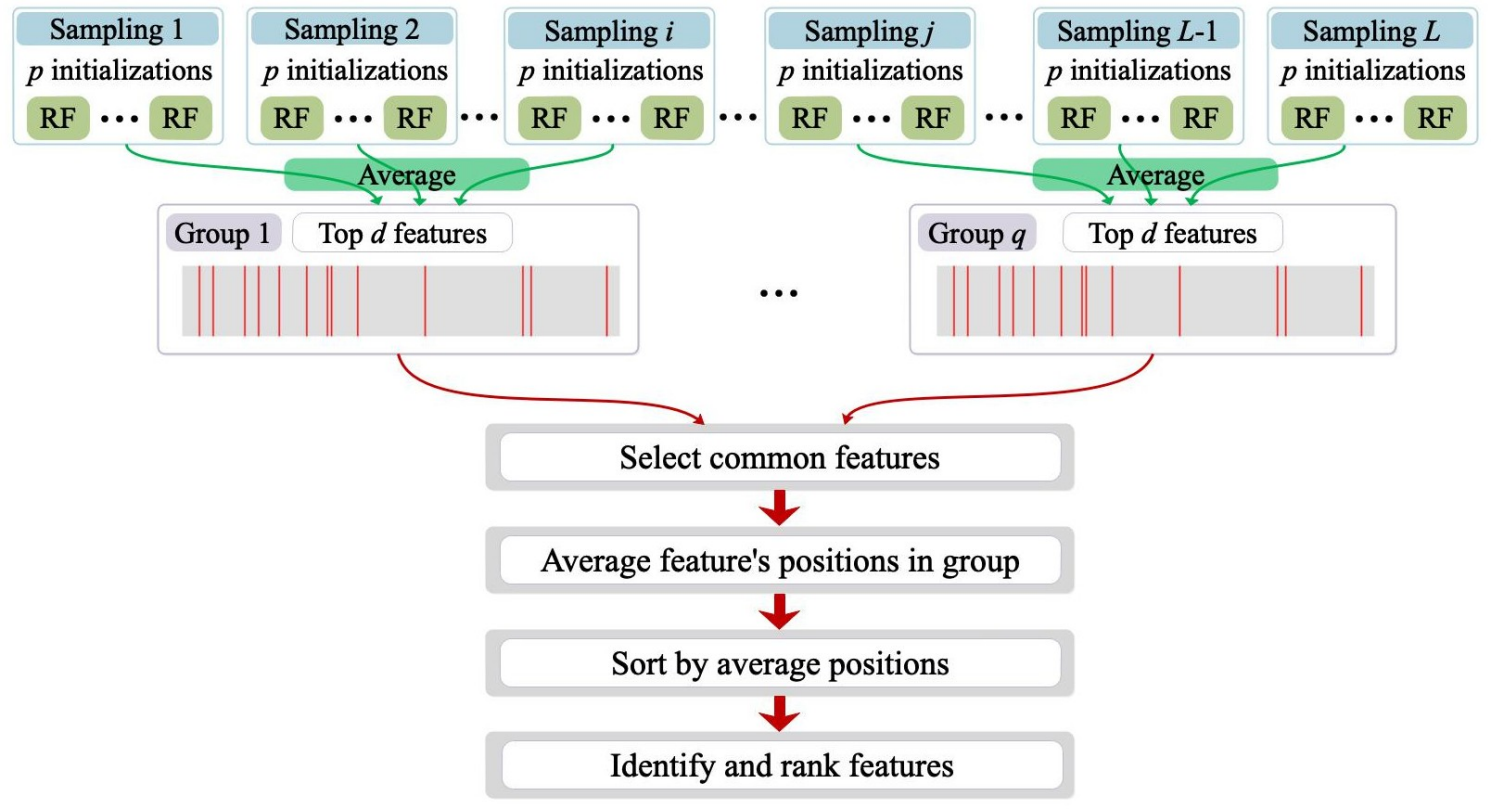
The procedure for calculation of feature importances from multiple RFs.

In order to guarantee the robustness and stability of the identified genes, for the calculation of feature importances from multiple RFs, we adopted a strategy of firstly grouping, then averaging, and finally intersecting; see Fig 2. First, we grouped the feature importances from the *L* samplings of bootstrapping step into *q* subgroups by averaging. Second, in each group, we identified the top *d* features. Third, we selected the common features of the *d* features from different groups. Fourth, we averaged the positions of these common features in their group. Finally, we obtained the identified and ranked features by sorting their average positions. The number of the resulting ranked features may be smaller than *d*, because features in the top *d* features from different subgroups may be different. Further, in order to make the selected features more stable and reduce the variation due to initialization, a number of *p* initializations were used in each sampling calculation. Besides, we theoretically analyzed or empirically examined the effects of the number of initializations *p*, number of sampling *L*, number of subgroups *q*, and number of top features in each group *d* on the performance of IMRF; we concluded that IMRF is consistent for these hyper-parameters; see the discussion in Section 8 in S1 File.

### Computational complexity

For RF, its time complexity is O(Tmnlogn) [13], where *T* is the number of decision trees, *n* is the number of samples, and *m* is the number of features. While using RF as a building block, our proposed IMRF requires *L* samplings and *p* initializations in each sampling calculation. So, the time complexity of IMRF is O(LpTmnlogn); that is, the time complexity of IMRF is about *Lp* times more than that of RF. However, it is worth noting that, with IMRF, it is easy to use multiple cores to parallelize, such as parallel computations for *Lp* RFs or parallel computations for different decision trees in these RFs. Thus, in practice, this parallelization can significantly reduce the runtime of IMRF, even rivaling RF.

### Invariance and variance of informative features

In the following, we give a simple yet important theorem to elucidate why sometimes the informative features/genes discriminative for more classes are not so for fewer classes, sometimes the features/genes are discriminative for fewer classes but not for more classes, and when there exist discriminative features/genes both for more classes and for fewer classes. Without loss of generality, we consider three different samples from three different classes; the proof is given in Section 1 in S1 File, and it can be easily generalized to more classes.

**Theorem 1** (Invariance and variance of informative features). *Let* $O_{i}\in R^{m}$, *i* = 1, 2, 3, *denote three different samples from three different classes*. *Let* $\phi :R^{m}\to \Omega^{k}$ *be a feature mapping. Let*

$$\text{Com}\left(O_{1,2,3}\right)≜\phi \left(O_{1}\right)\cap \phi \left(O_{2}\right)\cap \phi \left(O_{3}\right)$$

*and*

$$\text{Dis}\left(O_{1,2,3}\right)≜\phi \left(O_{1}\right)\cup \phi \left(O_{2}\right)\cup \phi \left(O_{3}\right)-\phi \left(O_{1}\right)\cap \phi \left(O_{2}\right)\cap \phi \left(O_{3}\right).$$

*That is*, Com(*O*_1,2,3_) *and* Dis(*O*_1,2,3_) *respectively represent the common features and the discriminating features of O*_1_, *O*_2_, *and O*_3_. *Then we have the following properties*:

1) *If ω* ∈ Dis(*O*_1,2,3_), *then ω* ∈ Dis(*O*_1,2_), *ω* ∈ Com(*O*_1,2_), *or ω* ∈ *ϕ*(*O*_3_);
2) *If ϕ*(*O*_1_) *and ϕ*(*O*_2_) *are distinct, i.e.*, Dis(*O*_1,2_) ≠ ∅, *then there exists a feature ω* ∈ Dis(*O*_1,2,3_), *such that*
  $$\omega \in \text{Dis}(O_{1,2});$$
3) #Dis(*O*_1,2_) ⩽ #Dis(*O*_1,2,3_);
4) *Further, suppose that we stratify the discriminated features into two levels*:
  $$\begin{array}{cc} & {\text{Dis}}^{l1}\left(O_{1,2,3}\right)\\ ≜ & \phi \left(O_{1}\right)\cup \phi \left(O_{2}\right)\cup \phi \left(O_{3}\right)-\left(\phi \left(O_{1}\right)\cap \phi \left(O_{2}\right)\right)\\ & \cup \left(\phi \left(O_{1}\right)\cap \phi \left(O_{3}\right)\right)\cup \left(\phi \left(O_{2}\right)\cap \phi \left(O_{3}\right)\right), \end{array}$$
  *and*
  $$\begin{array}{cc} & {\text{Dis}}^{l2}\left(O_{1,2,3}\right)\\ ≜ & \left(\phi \left(O_{1}\right)\cap \phi \left(O_{2}\right)\right)\cup \left(\phi \left(O_{1}\right)\cap \phi \left(O_{3}\right)\right)\\ & \cup \left(\phi \left(O_{2}\right)\cap \phi \left(O_{3}\right)\right)-\phi \left(O_{1}\right)\cap \phi \left(O_{2}\right)\cap \phi \left(O_{3}\right). \end{array}$$

*Generally, the features in* Dis^*l*1^(*O*_1,2,3_) *are considered more differentiating than those in* Dis^*l*2^(*O*_1,2,3_). *Then, we have the following properties*:

(1) Dis^*l*1^(*O*_1,2_) = Dis(*O*_1,2_);
(2) Dis^*l*1^(*O*_1,2,3_) ∩ Dis^*l*2^(*O*_1,2,3_) = ∅;
(3) Dis^*l*1^(*O*_1,2,3_) ∪ Dis^*l*2^(*O*_1,2,3_) = Dis(*O*_1,2,3_);
(4) *If*$\text{Com}\left(O_{1,2,3}\right)⫋\text{Com}\left(O_{1,3}\right)$, *or*$\text{Com}\left(O_{1,2,3}\right)⫋\text{Com}\left(O_{2,3}\right)$, *then there exists a feature ω, such that*
  $$\omega \in \text{Dis}(O_{1,2}),$$
  *but*
  $$\omega \notin {\text{Dis}}^{l1}\left(O_{1,2,3}\right).$$

## Results

### Datasets to be used

**Fig 3:**
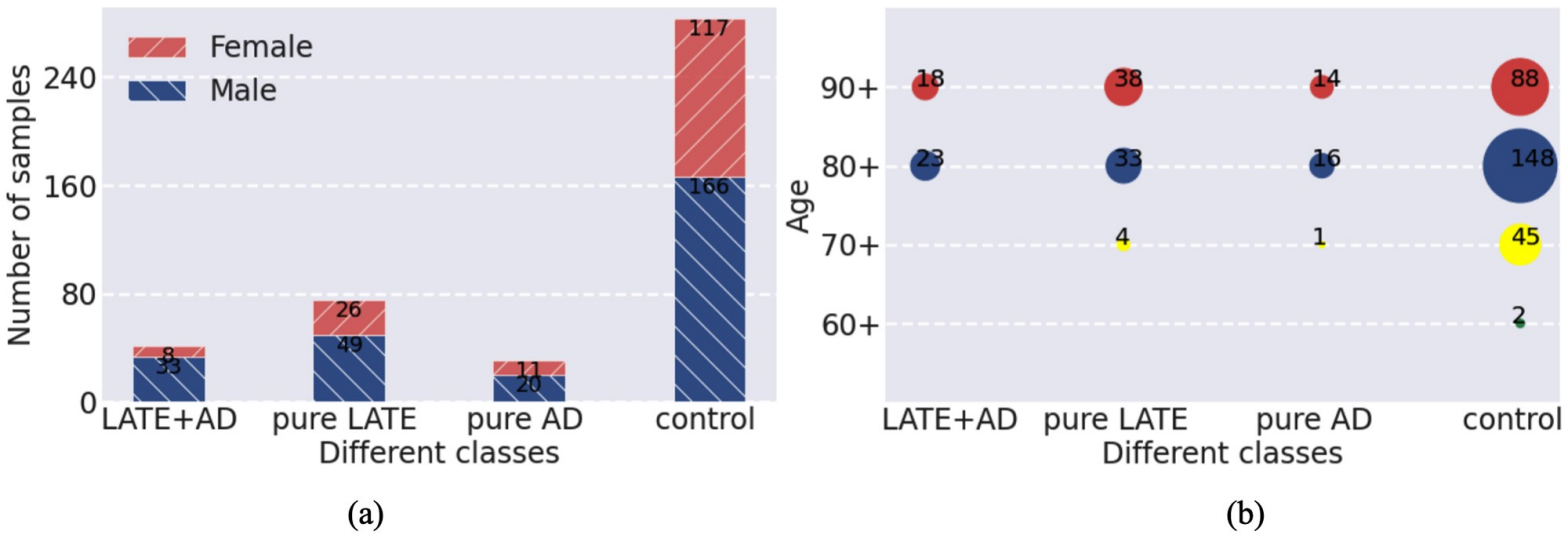
Demographics for the stratified study population of RNA array expression. (a) Distribution with respect to four classes, LATE+AD, pure LATE, pure AD, and control, in sex. The vertical axis represents the number of samples. (b) Age distribution with respect to the four classes. The vertical axis represents the age of samples. The horizontal axes for (a) and (b) denote different classes.

Gene expression datasets used in this study are from the Religious Orders Study and Memory and Aging Project (ROSMAP) (See link https://www.synapse.org/#!Synapse:syn3219045. This data consists of two parts. One is a longitudinal clinical-pathologic cohort study of aging and AD, and the other is a longitudinal, epidemiologic clinical-pathologic cohort study of common chronic conditions of aging with an emphasis on the decline in cognitive and motor function and risk of AD). The RNA array expression data for brain samples (With Synapse ID syn3800853) were obtained from the RADC research resource sharing hub (An AD research centers designated and funded by the National Institute on Aging. See link https://www.radc.rush.edu), and so were the corresponding clinical indexes and pathology annotations. RNA expression-label association was performed on the datasets. The original RNA array data contain 490 samples. After preprocessing, finally we obtained 430 samples, each with 48,803 features (The total number of different genes is 37,846. For the accuracy of the analysis, we did not preprocess the measurements for the same genes). We categorized these samples, with Braak and CERAD scores used for recognizing AD, and TDP-43 stage for LATE. The detailed rules for categorization are presented in Table 1. After categorizing, we obtained 41 samples for LATE+AD, 75 samples for pure LATE, 31 samples for pure AD, and 283 samples for control. We present the demographics for the study population stratified by these rules in Fig 3.

Besides, details on hyper-parameters of IMRF used in this study are provided in Section 2 in S1 File. Although our algorithm was developed for gene expression data, for assessing its effectiveness of selecting informative features with imbalanced general data, we used three additional datasets: one synthetic dataset, one cross-domain dataset (i.e., MNIST [14], which is from the computer vision field), and one AD RNA dataset from the Mount Sinai Brain Bank Array Tissue Panel Study (MSBB_ArrayTissuePanel) See link https://www.synapse.org/#!Synapse:syn3157699.

The synthetic and cross-domain datasets were used because they may provide a visually meaningful way for the validation of IMRF in both classification and feature identification with imbalanced multi-class data. MNIST has a training set of 60, 000 collected handwritten digits and a test set of 10, 000 examples, each digitized to a 28 × 28 grayscale image, and synthetic databast consists of 400 samples of size 112 × 92 which are generated by randomly sampling from interval [0, 244]. We considered the following task for the purpose of validating our algorithm: For MNIST, we chose 400 samples with the digits 1 and 9 by a ratio of 19:1, and we did the same with 3 and 8. These ratios are similar to those of different classes in ROSMAP data. For synthetic data, firstly, we added “artificial informative features” to images with a 4 × 4 black point on the upper left corner, the lower left corner, and both. Then, the resulting dataset has four classes, i.e., class 0 without any black point, class 1 with a black point on the upper left corner, class 2 with a black point on the lower left corner, and class 3 with black points on both upper and lower left corners (The black point on these corners is not fixed; instead, it is designed to randomly appear in four directions with an offset of 3 pixels. This design is to mimic the subtle variations that might occur in the location of genes). These four classes were designed to be at a ratio of 26:3:7:4. These ratios also mimiced those of the classes with ROSMAP data. In addition, we considered a cross case: there is a common black point in the middle of the right side for the above classes 1 and 2. For these two datasets with artificial features, we illustrate randomly chosen examples in Tables 1–4 in Section 3 of S1 File.

The prefrontal cortex (PFC) is responsible for high levels of cognitive function, including working memory and language. PFC with AD is prone to neurodegeneration [15]. So, we chose AD RNA data of prefrontal cortex from MSBB_ArrayTissuePanel as an additional source of data for further validation. The AD dataset contained 56 samples, each with 35,339 features after preprocessing. We annotated the samples into 39 controls and 17 ADs, where control samples are those devoid of AD neuropathological changes in the brain, with 〚Braak〛 < 5, and AD samples are those with extensive AD neuropathological changes in the brain, with 〚Braak〛 ⩾ 5.

### Validation on synthetic and cross-domain datasets

**Fig 4:**
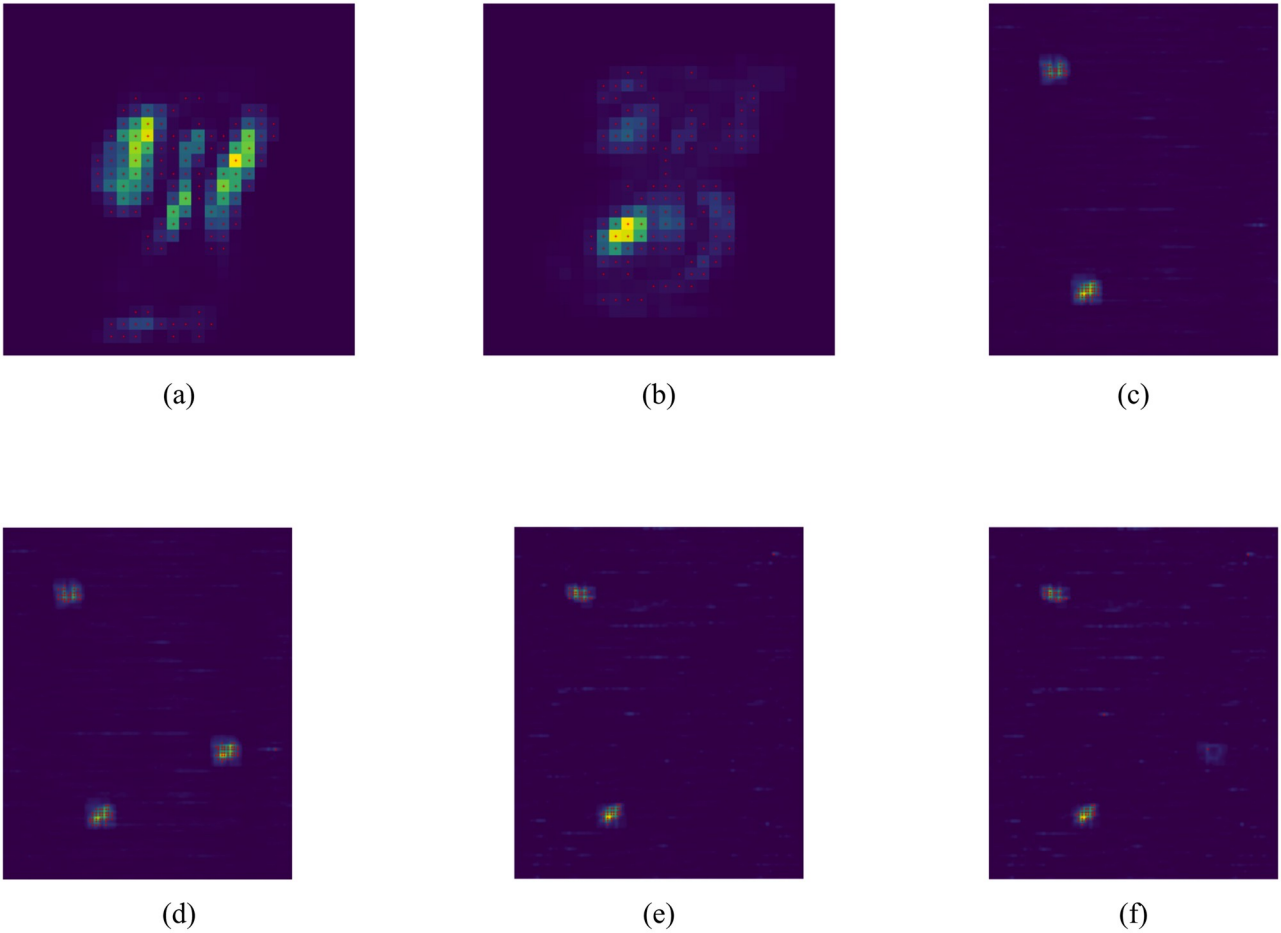
Supervised feature selection on MNIST and synthetic data. (a) MNIST with the digits 1 and 9; (b) MNIST with the digits 3 and 8; (c) Four classes of noise background images with or without black points; (d) Four classes of noise background images with or without cross black points. The black point in the middle of the right side is a common black point for classes 1 and 2; (e) Using classes 1 and 2 in Table 3 in Section 3 of S1 File for classification and feature selection; (f) Use classes 1 and 2 in Table 3 in Section 3 of S1 File for classification and feature selection. In (a)-(f), the selected features are marked in red for visualization. Best viewed with color when zoomed in.

Firstly, we validate IMRF on above-designed MNIST and synthetic datasets. The feature identification-related results are displayed for visual inspection in Fig 4, and the classification results are provided in the Section 4 in S1 File. It is observed that IMRF effectively locates and detects informative features, in spite of significant variations of different noise backgrounds. Interestingly, from Fig 4(e), it is observed that the samples with classes 1 and 2 have identified features similar to those with all four classes in Fig 4(c). On the other hand, from Fig 4(f), it is seen that the identified features in the middle of the right side in Fig 4(d) are not included in (f). The reason is that, if some important features for discriminating four classes are shared by classes 1 and 2, they would not be selected as important features for classifying classes 1 and 2. Further, Fig 4(e) and 4(f) both show that the classification for two classes has fewer identified features than those for four classes, implying that classifying two classes generally depends on fewer informative features than four classes. Additionally, in Fig 4(e) and 4(f), some features, which are not so informative for four classes, are identified as important for two-class scenarios. The above observations can be made more general, which have theoretical guarantees given in Theorem 1.

### Validation on AD dataset

We adopt IMRF to select the top 5 genes, which are shown in Table 2. It is seen that these genes are already discussed in prior AD studies.

**Table 2:** Top 5 genes identified and ranked from 35,339 genes for differentiating controls and ADs, and the related prior studies in the literature on these genes.

Rank	Gene name	Related study
1	*TGFBR3*	[16–18]
2	*MRC2*	[19]
3	*NFX1*	[20]
4	*RGS1*	[19, 21]
5	*LAMA2*	[22]

### Classification and gene identification

**Fig 5:**
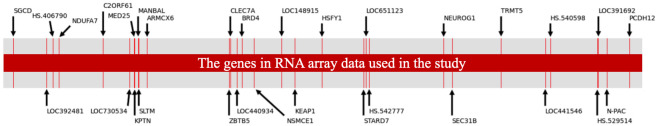
The 31 genes selected from 48,803 genes by IMRF. Red vertical lines with gene names represent the IMRF-identified genes.

The validation using the above synthetic cross-domain datasets and AD RNA dataset manifests that IMRF can effectively pinpoint important features, despite strong variations of noises in the background and high dimensionality of RNA data. Here, we applied it to the preprocessed AD and LATE brain transcriptome-wide data to classify samples and then identify the disease-associated genes. We respectively presented the classification results in Fig 3 and Table 9 in Section 5 of S1 File, and visually depicted the top 31 identified genes in Fig 5. Also, we ranked these top 31 genes and provided the existing studies related to them in Table 4. About half of these identified genes by IMRF were implicated with prior neurodegeneration and aging studies.

**Fig 6:**
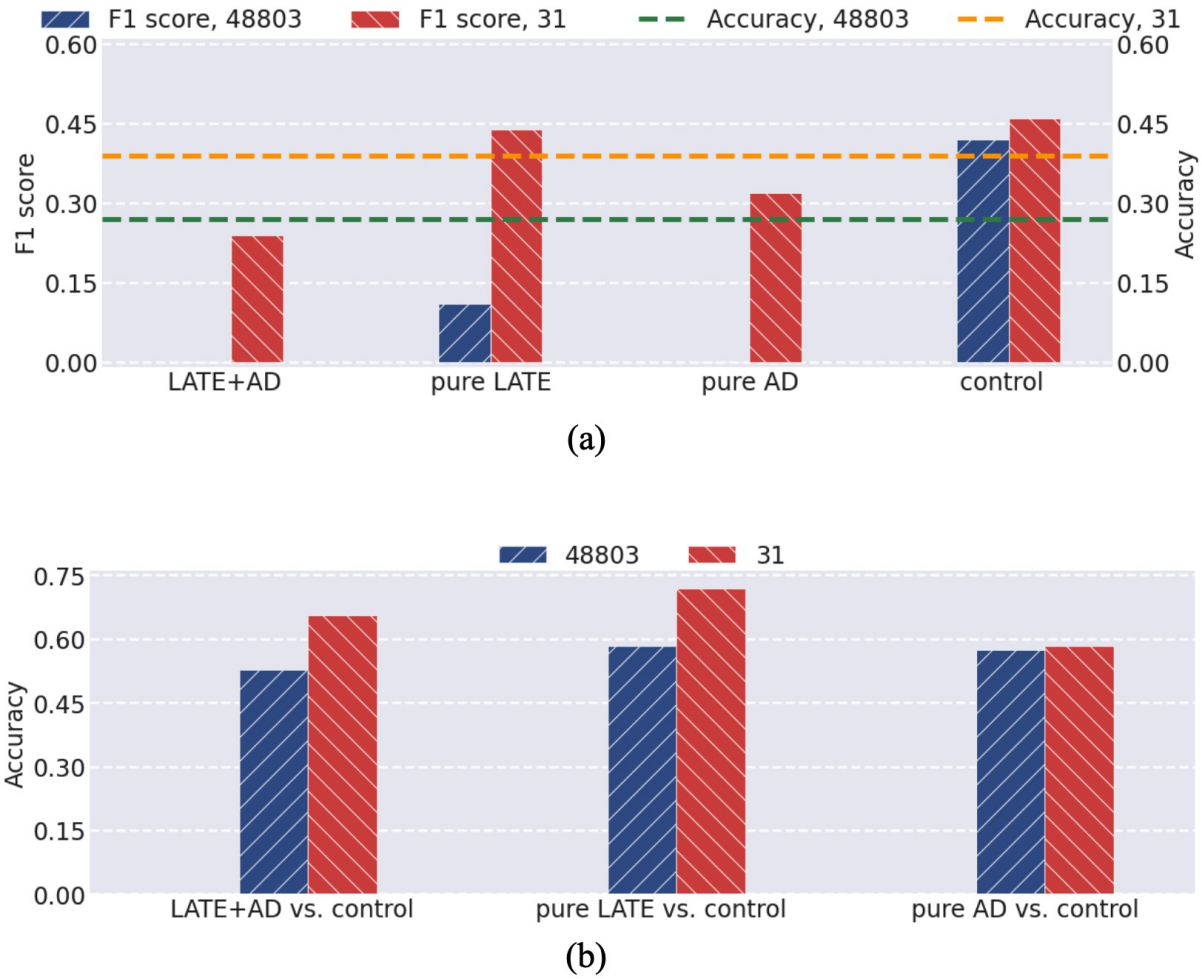
Comparison of F1 scores and accuracies by SVM on the total and IMRF-selected genes. (a) Class-wise F1 scores and overall accuracy for four-class classification; (b) Accuracy for three scenarios of binary classification.

For further verifying the significance of IMRF-identified genes, we performed the classification and gene identification for four-class classification and for binary classification with two different classes using IMRF. Moreover, for fairly comparing their performance, we used the Support Vector Machines (SVM) model with a polynomial kernel as a benchmark classifier, which is based on function approximation and thus completely different from rule-based decision trees and RF. We respectively applied it to the total genes and the IMRF-identified genes. Concretely, we studied the following three cases:

**Case 1**: We compared the performance of SVM on the IMRF-identified genes to that on the total genes, and the resulting precisions and accuracies are shown in Fig 6(a). It is evident that the performance on the IMRF-identified genes has been improved upon all the genes. Such a result implies that the subset of genes identified by IMRF is significant to distinguish LATE+AD, pure LATE, pure AD, and control, as independently verified by a totally different classifier from those used in IMRF.

**Case 2**: Based on those IMRF-identified genes in Case 1, we implemented SVM on the IMRF-identified genes and the total genes for three scenarios of binary classifications, including LATE+AD vs. control, pure LATE vs. control, and pure AD vs. control. The results are given in Fig 6(b). It is found that the performance on the IMRF-identified genes has been improved in all three scenarios, but the improvement in each scenario is not as large as that in Case 1; the reason is that the IMRF-identified genes are for all four classes and some of these genes become less important when classifying two classes, analogous to what was shown in (d)-(f) of Fig 4 for synthetic and cross-domain data.

**Fig 7:**
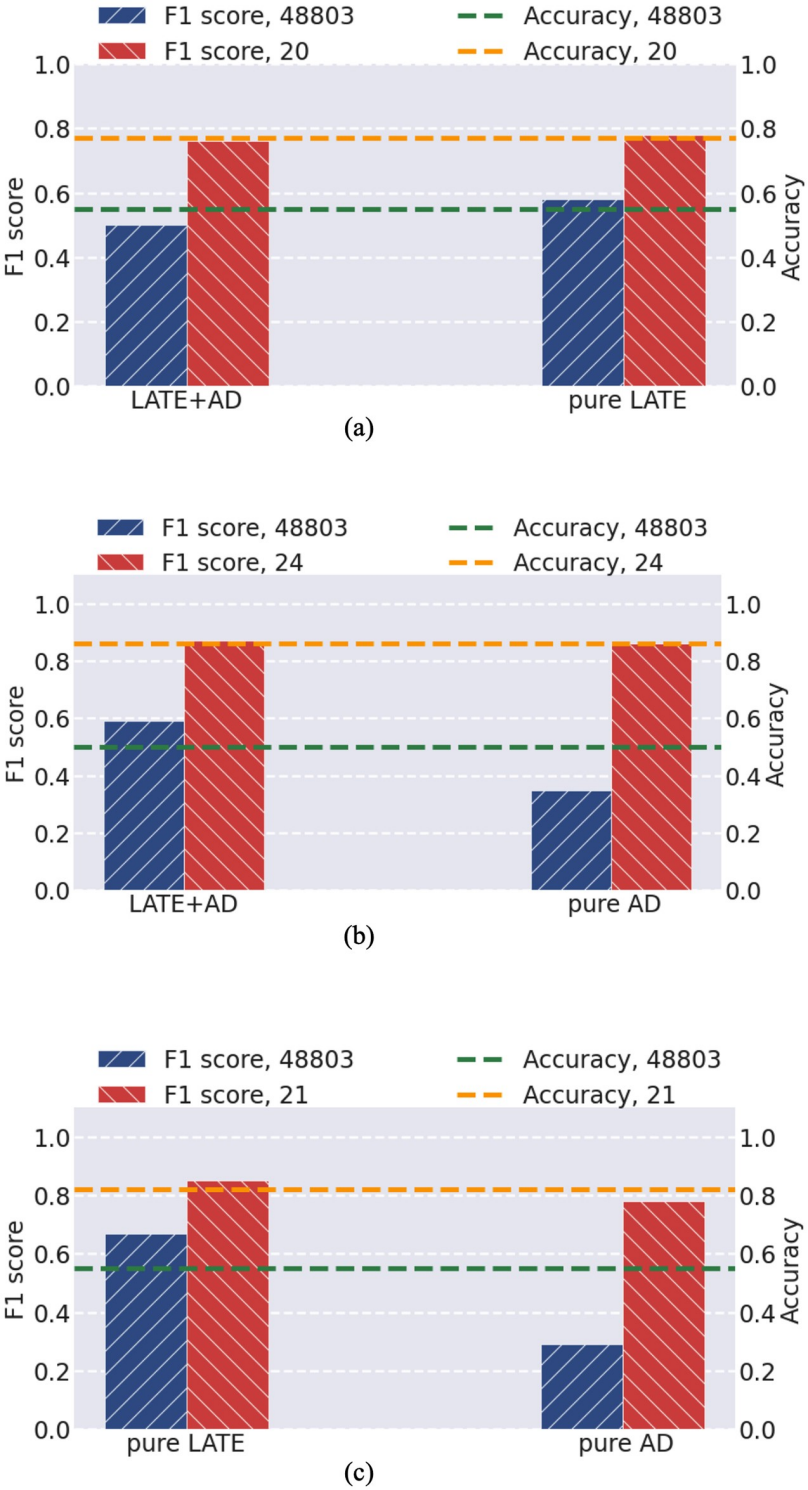
Comparison of F1 scores and accuracy for three scenarios of binary classification using the total genes and using the IMRF-selected genes. (a) LATE+AD vs. pure LATE; (b) LATE+AD vs. pure AD; (c) pure LATE vs. pure AD.

**Case 3**: We further considered the scenarios of classifying the remaining pair-wise classes in Case 2, including LATE+AD vs. pure LATE, LATE+AD vs. pure AD, and pure LATE vs. pure AD. We directly applied IMRF to find the informative genes for discriminating these pair-wise classes. Then we also adopted SVM to classify the IMRF-identified genes and the total genes. The results are depicted in Fig 7. It is apparent that for all scenarios of classifying these pair-wise classes, the performance on IMRF-identified genes is significantly improved upon all genes.

The IMRF-identified genes on all six pair-wise classes, including LATE+AD vs. pure LATE, LATE+AD vs. pure AD, pure LATE vs. pure AD, LATE+AD vs. control, pure LATE vs. control, and pure AD vs. control, are displayed in Table 3. It is noted that some of the IMRF-identified genes for binary classifications are also identified when classifying for four classes, as shown by Table 4, but some identified genes are different. This observation is similar to that for Case 2, as analogously shown in (d)-(f) of Fig 4 for synthetic and cross-domain data. These empirical results are theoretically proved to be true in Theorem 1.

**Table 3:** Genes identified by IMRF from 48803 genes for six scenarios of pair-wise classes. The p-values calculated by ANOVA are shown in the parentheses. The genes in bold are also selected for differentiating four classes, which are shown in Table 5. There are respectively 4, 6, 7, 1, 3, and 3 genes with p-values greater than 0.05 for LATE+AD vs. pure LATE, LATE+AD vs. pure AD, pure LATE vs. pure AD, LATE+AD vs. control, pure LATE vs. control, and pure AD vs. control.

Class	Gene name (p-value)
LATE+AD vs. pure LATE (20)	***HS.406790*** (1.02E-4), ***NDUFA7*** (7.91E-5), *HS.253475* (1.31E-3), *DDX26B* (5.20E-2), ***MANBAL*** (8.84E-1), *C8ORF58* (3.62E-4), *OVOS2* (9.51E-1), ***ZBTB5*** (9.75E-5), *VGF* (7.67E-5), *HS.559151* (1.07E-2), *HS.561747* (1.29E-4), ***KEAP1*** (6.66E-4), *HS.554346* (2.97E-3), ***STARD7*** (1.67E-4), ***LOC651123*** (4.38E-3), *UIMC1* (7.26E-2), ***SEC31B*** (2.91E-2), *HS.128396* (1.34E-3), ***LOC441546*** (7.62E-4), ***LOC391692*** (7.48E-5)
LATE+AD vs. pure AD (24)	*RNASE4* (8.83E-1), *OSR2* (2.72E-3), *EPGN* (1.57E-4), *CDC6* (2.18E-4), *SP140* (1.26E-1), *ADSSL1* (5.21E-1), *OVOS2* (2.71E-1), *LOC645723* (4.38E-3), ***CLEC7A*** (7.17E-1), *HS.543051* (7.59E-4), *HS.560742* (3.80E-5), *IL29* (2.89E-1), *LOC648251* (3.82E-3), *TNR* (1.69E-3), *TPSG1* (4.73E-2), *FGF16* (1.46E-3), *HS.416810* (1.48E-2), *HS.135067* (8.17E-3), *FBXO43* (2.00E-3), *HS.536734* (4.25E-3), *HS.156651* (3.78E-5), *PLA2G15* (2.21E-3), *FLJ42133* (3.36E-4), *BARX2* (1.22E-3)
pure LATE vs. pure AD (21)	*ANAPC11* (6.91E-2), ***SGCD*** (4.85E-1), *CDC6* (4.69E-4), *HYOU1* (5.56E-4), *GRIPAP1* (4.51E-2), *DTNB* (4.33E-2), *NIPBL* (5.00E-1), ***SLTM*** (2.59E-4), *XKRY* (3.50E-3), *ZHX1* (5.37E-3), *SEC14L5* (7.27E-4), ***CLEC7A*** (7.79E-1), *GOLGA4* (2.33E-3), *PSMB8* (9.93E-1), *USP4* (7.95E-1), *ZNF823* (2.24E-3), *FBXO43* (1.18E-4), *SRPR* (4.05E-5), *HS.581994* (1.14E-3), *INHA* (1.20E-2), *BHLHB9*(8.37E-2)
LATE+AD vs. control (12)	***NDUFA7*** (2.75E-7), *LOC644291* (6.02E-4), *DDIT3* (6.68E-3), ***LOC730534*** (5.31E-6), ***MED25*** (1.52E-5), *HSP90B1* (3.97E-5), ***NSMCE1*** (7.01E-5), ***LOC148915*** (2.59E-7), *SDSL* (5.72E-4), *NRIP2* (1.28E-6), *SMAD7* (5.75E-1), *SLC6A12* (5.39E-7)
pure LATE vs. control (18)	***SEC31B*** (3.46E-3), ***LOC392481*** (7.24E-5), ***NEUROG1*** (8.55E-5), ***N-PAC*** (3.83E-5), ***HS.540598*** (7.54E-5), ***SGCD*** (7.38E-1), *HS.543684* (9.86E-5), ***HS.542777*** (6.39E-5), ***C2ORF61*** (9.00E-4), *HS.545899* (4.10E-3), *RBM4* (1.99E-1), *LOC150207* (1.59E-4), *AHCTF1* (5.51E-2), *ARF1* (7.62E-3), *HS.579437* (3.48E-3), *TMSB4X* (4.15E-4), *HS.549460* (2.55E-3), ***HSFY1*** (1.80E-3)
pure AD vs. control (14)	*ALG9* (8.66E-5), *CDC6* (4.38E-4), *C11ORF17* (3.10E-1), *LOC392395* (6.34E-4), *JUB* (4.58E-1), *ALAD* (3.04E-4), *HS.581468* (4.22E-3), *HS.543116* (5.71E-3), *LOC651208* (7.81E-3), ***CLEC7A*** (7.69E-1), ***LOC440934*** (2.59E-3), *LOC728056* (3.06E-4), *SEPHS1* (1.86E-3), *INHA* (2.94E-2)

**Table 4:** Top 31 genes identified and ranked from 48803 genes for differentiating the four classes, their p-values by using ANOVA, and the related studies on these genes.

Rank	Gene name	p-value	Related study
1	*LOC391692*	1.09E-6	
2	*NEUROG1*	9.79E-4	AD [24]
3	*STARD7*	6.84E-5	AD [25]
4	*LOC148915*	2.47E-7	
5	*CLEC7A*	7.12E-1	Neurodegenerative diseases [26–29]
6	*SEC31B*	2.83E-2	AD [30]
7	*MED25*	5.37E-6	
8	*SGCD*	6.74E-1	AD [31] and limb girdle muscular dystrophies [32]
9	*N-PAC*	2.34E-4	Neurodegenerative diseases [33]
10	*KPTN*	3.72E-5	AD [34, 35]
11	*HS.529514*	4.86E-6	
12	*LOC392481*	3.69E-4	
13	*LOC441546*	7.00E-4	
14	*MANBAL*	1.78E-1	
15	*HS.542777*	7.76E-5	
16	*LOC730534*	8.67E-6	
17	*NDUFA7*	8.32E-7	AD [34, 36] and other types of dementia [37]
18	*C2ORF61*	2.82E-3	
19	*HS.540598*	1.58E-3	
20	*KEAP1*	1.42E-3	AD [38–42]
21	*LOC440934*	2.65E-4	
22	*TRMT5*	7.21E-6	Its mutations will cause exercise intolerance, neuropathy, and muscle weakness or developmental delay and spastic paraparesis [43]
23	*ARMCX6*	1.38E-1	Associated to mental retardation syndromes but with unknown molecular basis [44]
24	*BRD4*	9.90E-1	Cognition and memory [45, 46]
25	*HS.406790*	7.50E-4	
26	*PCDH12*	6.00E-2	Brain calcifications [47]
27	*NSMCE1*	2.56E-4	Involved in maintaining genome integrity, DNA damage response, and DNA repair. Defective DNA repair may lead to neurological disorders like AD [48]
28	*LOC651123*	5.53E-2	
29	*SLTM*	9.12E-5	AD [49, 50]
30	*ZBTB5*	2.57E-3	AD [51]
31	*HSFY1*	1.29E-2	The *APOE* genotypes are associated with *HSFY1* [52]

**Table 5:** Subject categorization rules for RNA expression data. Here, 〚⋅〛 denotes the grade corresponding to the specific metric.

Rule	Class
〚Braak〛 ⩾ 5, 〚CERAD〛 ⩽ 2, and 〚TDP-43〛 = 1	LATE+AD
〚Braak〛 < 5 or 〚CERAD〛 > 2, and 〚TDP-43〛 = 1	pure LATE
〚Braak〛 ⩾ 5, 〚CERAD〛 ⩽ 2, and 〚TDP-43〛 = 0	pure AD
〚Braak〛 < 5 or 〚CERAD〛 > 2, and 〚TDP-43〛 = 0	control

Additionally, it is worth noting that it is the first time/algorithm that aims to identify disease-specific genes and then classify LATE from AD. The classification accuracy based on IMRF-identified genes appears low, since LATE is a newly discovered disease and it mimics AD. And it is a highly challenging task; in particular, so far there has been no clinical biomarker to distinguish between the two diseases.

### Comparison of different algorithms

**Fig 8:**
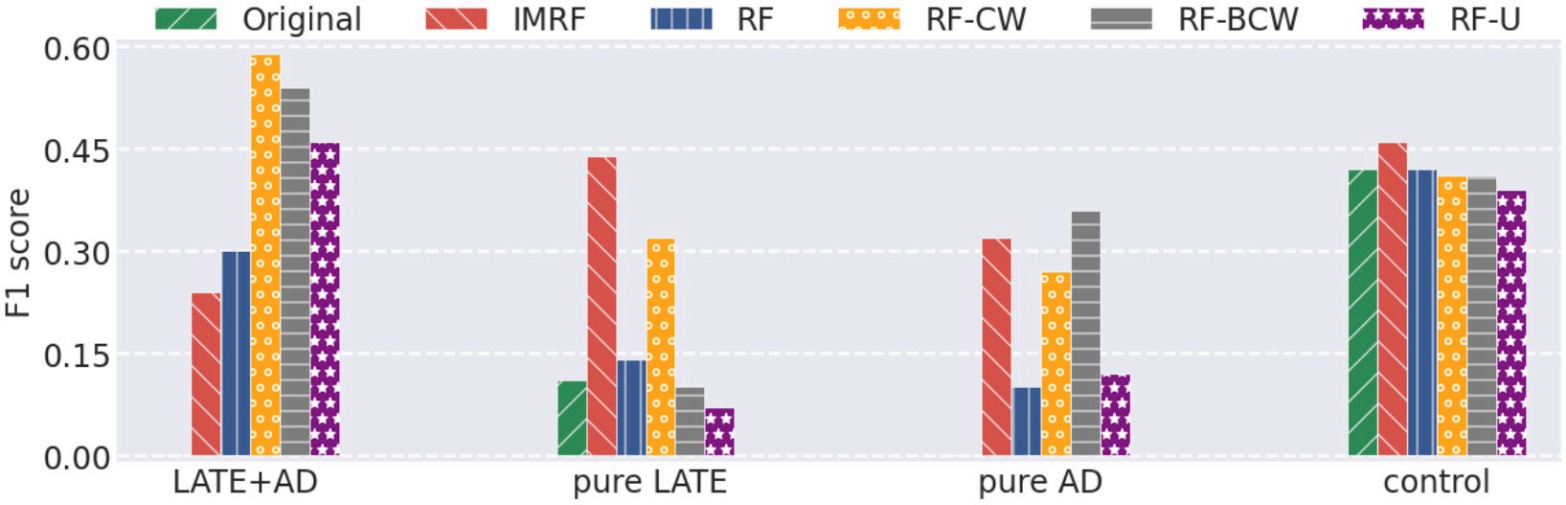
SVM classification performance in F1 score using the original number of genes and using the selected genes by different RF-based algorithms.

**Fig 9:**
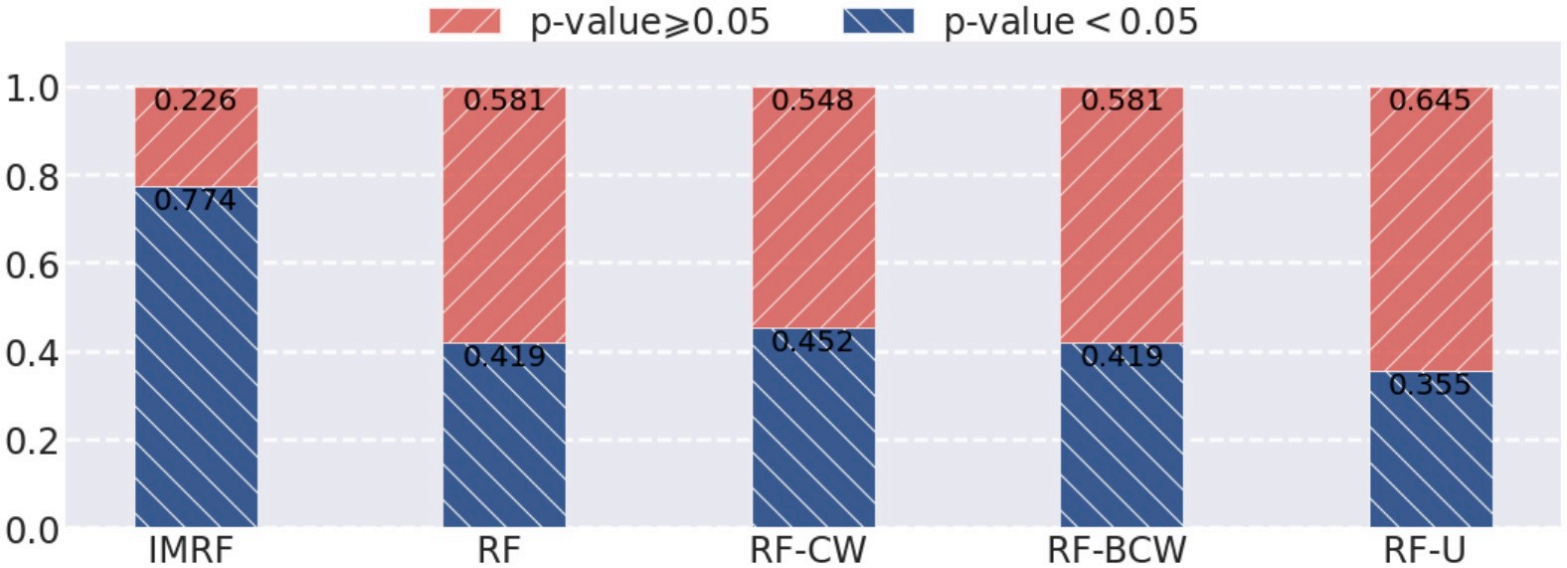
The ratios of genes with p-value ⩾ 0.05 vs. p-value < 0.05 for 31 selected genes by different algorithms.

To further demonstrate the effectiveness of IMRF, we compared it with RF-CW, RF-BCW, and RF-U on gene expression data. We used a baseline classification model SVM to evaluate the quality of the sets of identified genes by different RF-based algorithms. From each class we randomly chose 16 as test samples. For fair comparison, the numbers of trees and identified features were set to 10, 000 and 31, respectively for all algorithms in comparison. The results in F1 score were shown in Fig 8. The results, in precision and recall, and the identified genes by different algorithms were given in Fig 4 in Section 6 of S1 File. One can observe that IMRF achieves a more stable and class-balanced performance than other methods. Also, we provided the identified genes from different algorithms in Table 10 in Section 7 of S1 File, and we respectively compared the ratios of genes with p-value ⩾ 0.05 and p-value < 0.05 over 31 selected genes by different algorithms in Fig 9. It is observed that nearly 80% of IMRF-selected genes have p-values greater than 0.05, which is much higher than the other four algorithms.

**Fig 10:**
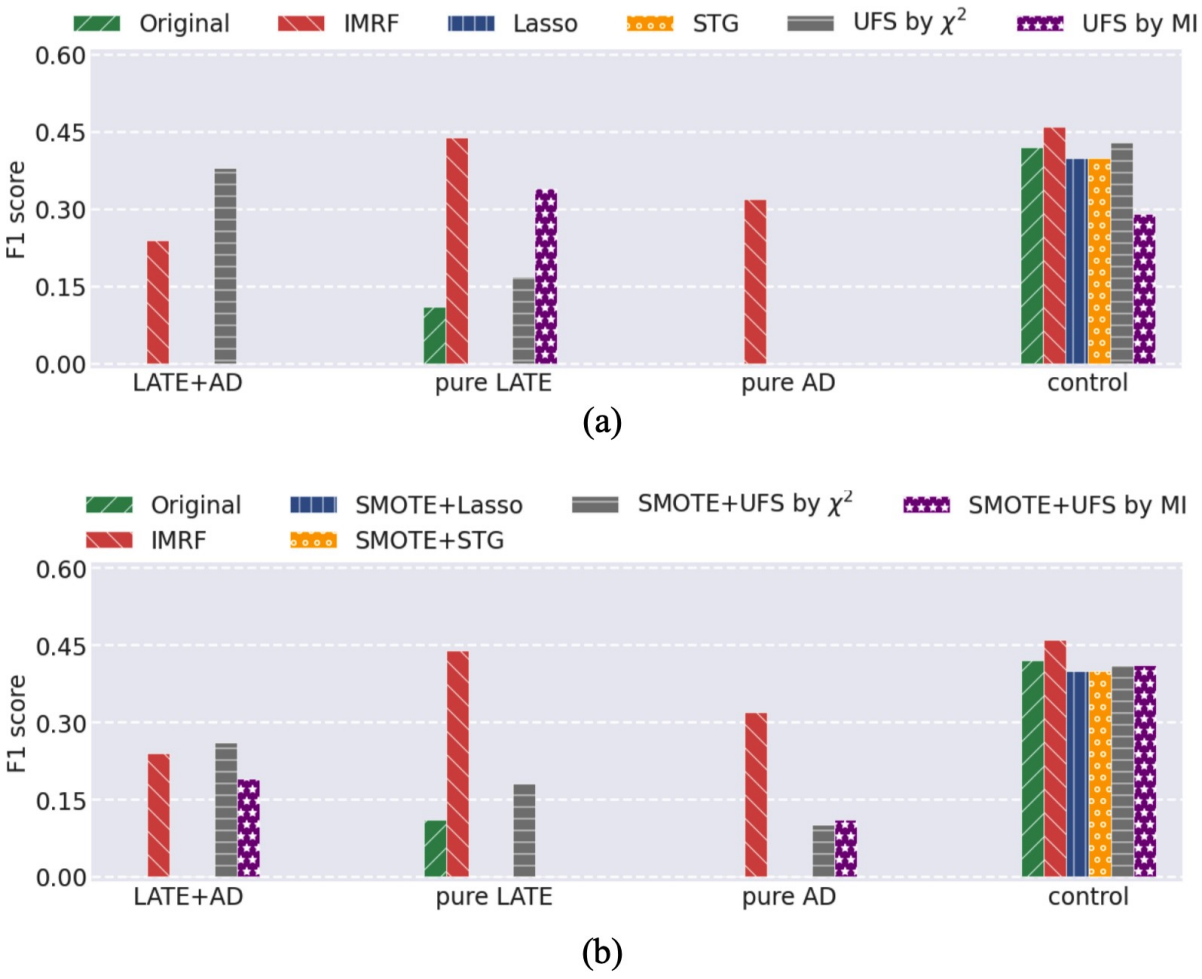
SVM classification performance in F1 score on the original number of genes and the selected genes by different feature selection algorithms. Without (a) or with (b) using SMOTE as a preprocessing procedure to counteract the class imbalance.

Besides, we compared IMRF with several existing feature selection algorithms: 1) STG, which is based on probabilistic relaxation of the *ℓ*_0_ norm for feature selection; 2) Lasso, which is by *ℓ*_1_ norm to select features; 3) UFS, which was adopted from scikit-learn, where two filters were considered, that is, *χ*^2^ test and mutual information (MI). Meanwhile, we also considered adopting SMOTE [23] as a preprocessing for these algorithms. The results in F1 score were provided in Fig 10, indicating that IMRF achieved a superiority performance over these algorithms.

## Discussion

We used IMRF to identify 31 genes with disease-related differential expression (out of 48803 genes). By ranking these genes, using ANOVA to calculate the p-value of each IMRF-selected gene, and relating them to prior neurodegeneration and aging studies in Table 4, we demonstrated that IMRF was effective at identifying informative genes potentially associated with neurodegenerative diseases. Among these 31 genes, at least 12 genes have already been related to neurodegenerative diseases in prior studies, with 10 being implicated with AD. The 22nd ranked gene *TRMT5* was found to affect motor intolerance and neuropathy, leading to muscle weakness, growth retardation, and spastic paraparesis [43]. The 23rd and 24th ranked genes, *ARMCX6* and *BRD4*, were linked to impairments in cognition and memory [44–46], which are regarded as the common symptoms of dementia. The 26th ranked gene *PCDH12* was previously associated with brain calcifications [47], which could cause memory loss, personality changes, and diminished intellectual function [53], thereby potentially leading to psychosis or neurocognitive disorder [54, 55]. The 31st ranked gene *HSFY1* was found to affect APOE4 genotypes, while the patients with different APOE4 genotypes, such as APOE4-negative and APOE4-positive, possibly have different decline speeds on language, attention, executive, and visuospatial functioning [56]. Though about half of the top-ranked genes were already implicated in neuropathology such as AD by prior studies in the literature, to the best of our knowledge, the remaining genes have not been reported for associations with neurodegenerative diseases.

**Fig 11:**
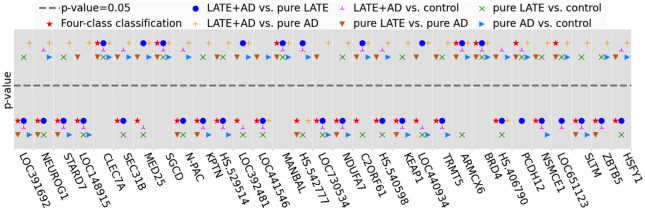
Schematic representation of the p-values of the IMRF-selected genes for four classes and six pair-wise classes.

We respectively compared the p-values of IMRF-selected genes for four classes and six pair-wise classes in Fig 11. It is seen that there are 7 genes having p-values greater than 0.05; only 4 genes for all cases with p-values greater than 0.05. Since the calculation of the p-value by ANOVA is for testing univariate and linear relationships, it does not consider the complex nonlinear feature-class relationships and interactions among features. In contrast, IMRF identifies genes by taking into account nonlinear relationships and interactions among different features. Thus, it is possible that some IMRF-selected genes individually and linearly have no significant effect on the disease, but may have a nonlinear effect on the disease or interact with other genes to have an effect on the disease.

Comparing Table 3 with Table 4, where the IMRF-selected genes were selected for differentiating four classes and for six pair-wise classes, respectively, one can observe that a number of genes identified for four classes are not among those selected for two classes. Yet, many are among those selected both for four classes and for two classes, including *HS.406790*, *NDUFA7*, *MANBAL*, *ZBTB5*, *KEAP1*, *STARD7*, *LOC651123*, *SEC31B*, *LOC441546*, and *LOC391692* for discriminating LATE+AD vs. pure LATE, with 5 of them already implicated in AD in prior studies; *CLEC7A* for LATE+AD vs. pure AD; *SGCD*, *SLTM*, and *CLEC7A* for pure LATE vs. pure AD, with the first two already associated with AD in prior studies; *NDUFA7*, *LOC730534*, *MED25*, *NSMCE1*, and *LOC148915* for LATE+AD vs. control, with *NDUFA7* and *NSMCE1* already linked to AD; *SEC31B*, *LOC392481*, *NEUROG1*, *N-PAC*, *HS.540598*, *SGCD*, *HS.542777*, *C2ORF61*, and *HSFY1* for pure LATE vs. control, with *N-PAC* related to other kinds of neurodegenerative diseases, and with *NEUROG1*, *SEC31B*, and *SGCD* previously associated to AD; and finally, *CLEC7A* and *LOC440934* for pure AD vs. control. Notably, *CLEC7A* is also highly ranked for LATE+AD vs. pure AD and LATE vs. pure AD, which was implicated in neurodegenerative diseases in prior studies. By Property 1) of Theorem 1, certain informative features for differentiating more classes may be not so informative for fewer classes. It explains why only a fraction of IMRF-selected genes for discriminating four classes are among those for pair-wise classes. By Property 2) of Theorem 1, as long as the samples in different pair-wise classes are distinct, one can always find important features that are simultaneously discriminative for more classes and for fewer classes. This property explains why there are always genes which are identified for two classes as well as for four classes. Finally, by Property 4) of Theorem 1, certain informative features that are able to differentiate fewer classes may fail to work for more classes. This property explains our observations that some genes highly ranked for discriminating two classes are not among those for four classes.

We have demonstrated that IMRF is effective to identify differentiating genes associated with AD and LATE based on the following evidences:

**Evidence 1**: Validation using synthetic and cross-domain datasets. IMRF can effectively detect differentiating features on synthetic and cross-domain datasets despite the strong interference from various backgrounds, as demonstrated in Fig 4;

**Evidence 2**: Cross-validation classification results on validation data sets. As shown in Table 9 in Section 5 of S1 File, IMRF achieves reasonably good performance for four-class classification on ROSMAP dataset;

**Evidence 3**: Enhanced classification performance using IMRF-identified genes compared with using all genes. Figs 6 and 7 reveal that the performance of a downstream classifier, SVM, which is completely independent from IMRF, using IMRF-identified genes has been obviously improved upon using all genes;

**Evidence 4**: 17 out of 31 selected genes were already implicated in neuropathology, such as AD and LATE, in prior studies. These genes were found to be closely linked to various types of neurodegenerative diseases.

In summary, IMRF-selected genes are promising for discriminating LATE, AD, and LATE+AD based on transcriptome-wide gene expression patterns; in particular, the remaining IMRF-identified genes in Table 4 that have not been reported in existing studies potentially warrant further study.

## Conclusion

IMRF enabled effective identification of putative genes associated with subjects having LATE and/or AD by discriminating them from controls based on transcriptome-wide data. Various forms of validations, such as verification on synthetic and cross-domain datasets, improved and competitive performance using the identified genes, testing the selected genes with a classifier that is completely independent from decision trees and RF, and relationships with prior studies on the genes linked to neurodegeneration, all testify to the effectiveness of IMRF in identifying genes with altered expression in LATE and/or AD. We conclude that IMRF is an algorithm of potential to facilitate the development of new gene biomarkers and targets for effective disease prevention and treatment strategies.

## Supporting information

S1 File Supplementary material of “Random forest-integrated analysis in AD and LATE brain transcriptome-wide data to identify disease-specific gene expression”. (PDF)
