# Supplementary material for: Random forest-integrated analysis in AD and LATE brain transcriptome-wide data to identify disease-specific gene expression
Source: PLoS One. 2021 Sep 7;16(9):e0256648. doi: 10.1371/journal.pone.0256648 (PMC8423259; doi:10.1371/journal.pone.0256648)
Supplement: S1 File — (PDF) [file pone.0256648.s001.pdf]

Supplementary Material of "Random forest-integrated analysis in AD and LATE brain transcriptome-wide data to identify disease-specific gene expression"

Xinxing Wu
University of Kentucky
Lexington, Kentucky, United States

Chong Peng
Qingdao University
Qingdao, Shandong, China

Peter T. Nelson
University of Kentucky
Lexington, Kentucky, United States

Qiang Cheng*
University of Kentucky
Lexington, Kentucky, United States

June 13, 2021

1 Proof of Theorem 1 2

2 Hyper-parameters for IMRF 5

3 Design of synthetic and cross-domain datasets 6

4 Classification results for synthetic and cross-domain datasets 7

5 Classification results for RNA expression data 10

6 Comparison of different algorithms 11

7 Identified genes from different algorithms 12

8 Hyper-parameter sensitivity analysis for IMRF 13

9 Codes 15

*Corresponding author. E-mail: qiang.cheng@uky.edu.

1 Proof of Theorem 1

Theorem 1 (Invariance and variance of informative features)**.** *Let $O_i \in \mathbb{R}^m$, $i = 1, 2, 3$, denote three different samples from three different classes. Let $\phi : \mathbb{R}^m \to \Omega^k$ be a feature mapping. Let*

$$\mathrm{Com}(O_{1,2,3}) \triangleq \phi(O_1) \cap \phi(O_2) \cap \phi(O_3),$$

and

$$\mathrm{Dis}(O_{1,2,3}) \triangleq \phi(O_1) \cup \phi(O_2) \cup \phi(O_3) - \phi(O_1) \cap \phi(O_2) \cap \phi(O_3).$$

That is, $\mathrm{Com}(O_{1,2,3})$ and $\mathrm{Dis}(O_{1,2,3})$ respectively represent the common features and the discriminating features of O_1, O_2, and O_3. Then we have the following properties:

1) If $\omega \in \mathrm{Dis}(O_{1,2,3})$, then $\omega \in \mathrm{Dis}(O_{1,2})$, $\omega \in \mathrm{Com}(O_{1,2})$, or $\omega \in \phi(O_3)$;

2) If $\phi(O_1)$ and $\phi(O_2)$ are distinct, i.e., $\mathrm{Dis}(O_{1,2}) \neq \emptyset$, then there exists a feature $\omega \in \mathrm{Dis}(O_{1,2,3})$, such that $\omega \in \mathrm{Dis}(O_{1,2})$;

3) $\#\mathrm{Dis}(O_{1,2}) \leqslant \#\mathrm{Dis}(O_{1,2,3})$;

4) Further, suppose that we stratify the discriminated features into two levels:

$$\mathrm{Dis}^{l1}(O_{1,2,3}) \triangleq \phi(O_1) \cup \phi(O_2) \cup \phi(O_3) - (\phi(O_1) \cap \phi(O_2)) \cup (\phi(O_1) \cap \phi(O_3)) \cup (\phi(O_2) \cap \phi(O_3)),$$

and

$$\mathrm{Dis}^{l2}(O_{1,2,3}) \triangleq (\phi(O_1) \cap \phi(O_2)) \cup (\phi(O_1) \cap \phi(O_3)) \cup (\phi(O_2) \cap \phi(O_3)) - \phi(O_1) \cap \phi(O_2) \cap \phi(O_3).$$

Generally, the features in $\mathrm{Dis}^{l1}(O_{1,2,3})$ are considered more differentiating than those in $\mathrm{Dis}^{l2}(O_{1,2,3})$. Then, we have the following properties:

(1) $\mathrm{Dis}^{l1}(O_{1,2}) = \mathrm{Dis}(O_{1,2})$;

(2) $\mathrm{Dis}^{l1}(O_{1,2,3}) \cap \mathrm{Dis}^{l2}(O_{1,2,3}) = \emptyset$;

(3) $\mathrm{Dis}^{l1}(O_{1,2,3}) \cup \mathrm{Dis}^{l2}(O_{1,2,3}) = \mathrm{Dis}(O_{1,2,3})$;

(4) If $\mathrm{Com}(O_{1,2,3}) \subsetneqq \mathrm{Com}(O_{1,3})$, or $\mathrm{Com}(O_{1,2,3}) \subsetneqq \mathrm{Com}(O_{2,3})$, then there exists a feature ω, such that $\omega \in \mathrm{Dis}(O_{1,2})$, but $\omega \notin \mathrm{Dis}^{l1}(O_{1,2,3})$.

Proof. 1) By condition $\omega \in \mathrm{Dis}(O_{1,2,3})$, we have

$$\omega \in \phi(O_1) \cup \phi(O_2) \cup \phi(O_3) - \phi(O_1) \cap \phi(O_2) \cap \phi(O_3).$$

If $\omega \notin \phi(O_3)$, then we have

$$\omega \in \phi(O_1) \cup \phi(O_2), \text{ but } \omega \notin \phi(O_1) \cap \phi(O_2);$$

or,

$$\omega \in \phi(O_1) \cap \phi(O_2).$$

So it is proved.

2) We prove it by reductio ad absurdum. We assume that, for any $\omega \in \mathrm{Dis}(O_{1,2,3})$, $\omega \notin \mathrm{Dis}(O_{1,2})$, i.e.,

$$\omega \notin \phi(O_1) \cup \phi(O_2) - \phi(O_1) \cap \phi(O_2). \tag{1}$$

Because of (1), we have two cases:

$$\omega \in \phi(O_3) - \phi(O_1) \cap \phi(O_2),$$

and

$$\omega \in \phi(O_1) \cap \phi(O_2).$$

By analyzing these cases, we obtain

$$\phi(O_1) = \phi(O_2);$$

that is,

$$\mathrm{Dis}(O_{1,2}) = \emptyset.$$

This obviously contradicts the given condition.

3) It is easily proved by the following facts:

$$\phi(O_1) \cup \phi(O_2) \subset \phi(O_1) \cup \phi(O_2) \cup \phi(O_3),$$

and

$$\phi(O_1) \cap \phi(O_2) \cap \phi(O_3) \subset \phi(O_1) \cap \phi(O_2).$$

4) By definition, (1), (2), and (3) can be easily proved. For (4), we prove it by reductio ad absurdum. We assume that, for any $\omega \in \mathrm{Dis}(O_{1,2})$, the following holds:

$$\omega \in \mathrm{Dis}^{l1}(O_{1,2,3}).$$

Note that $\mathrm{Com}(O_{1,2,3}) \subsetneqq \mathrm{Com}(O_{1,3})$, so we have

$$\mathrm{Com}(O_{1,3}) - \mathrm{Com}(O_{1,2,3}) \neq \emptyset.$$

By definition, we get

$$\mathrm{Com}(O_{1,3}) - \mathrm{Com}(O_{1,2,3}) \subseteq \phi(O_1).$$

Then we obtain

$$\mathrm{Dis}(O_{1,2}) \cap (\mathrm{Com}(O_{1,3}) - \mathrm{Com}(O_{1,2,3})) \neq \emptyset;$$

That is, there exists a feature ω_0, such that

$$\omega_0 \in \mathrm{Dis}(O_{1,2}),$$

and

$$\omega_0 \in \mathrm{Com}(O_{1,3}) - \mathrm{Com}(O_{1,2,3}).$$

Note that

$$\mathrm{Com}(O_{1,3}) - \mathrm{Com}(O_{1,2,3}) = \phi(O_1) \cap \phi(O_3) - \phi(O_1) \cap \phi(O_2) \cap \phi(O_3).$$

So, we have

$$(\mathrm{Com}(O_{1,3}) - \mathrm{Com}(O_{1,2,3})) \cap \mathrm{Dis}^{l1}(O_{1,2,3}) = \emptyset,$$

which implies that there exists a feature ω_0, such that

$$\omega_0 \in \mathrm{Dis}(O_{1,2}), \text{ but } \omega_0 \notin \mathrm{Dis}^{l1}(O_{1,2,3}).$$

If $\mathrm{Com}(O_{1,2,3}) \subsetneqq O_{2,3}^{\mathrm{Com}}$, similarly we come to the same conclusion.

Both contradict our assumption. Thus, it completes the proof. □

For the properties 1), 2), and 4) (4), they all have been empirically verified in the experiments on the synthetic and cross-domain datasets MNIST and noise background data; see Figure 4. Property 1) reveals that some informative features discriminative for more classes are not so for fewer classes, as evidenced by Figure 4 (d) and (f); Property 2) indicates that there exist informative features discriminative for more classes as well as for fewer classes, for example, Figure 4 (c) and (e); Property 4)(4) shows that there can be some informative features that are discriminative for fewer classes but are not so for more classes, as shown in Figure 4 (c) and (e).

2 Hyper-parameters for IMRF

In this study, we used the following hyper-parameters in IMRF: on MNIST, in the bootstrapping stage, we sampled the samples with labels 1 and 9, and with labels 3 and 8 at a ratio of 90:20, respectively. The number of trees was set to 150, the number of initializations for each sampling $p = 100$, the number of sampling $L = 100$, the number of subgroups $q = 4$, and the number of top features in each group $d = 150$. On synthetic data, for binary classification, in the bootstrapping stage, we sampled the samples with labels 1 and 2 at a ratio of 70:70. The number of trees was set to $2,100$, the number of initializations for each sampling $p = 100$, the number of sampling $L = 100$, the number of subgroups $q = 4$, and the number of top features in each group $d = 70$; for four-class classification, in the bootstrapping stage, we sampled the samples with labels 0, 1, 2, and 3 at a ratio of 90:30:70:40. The remaining parameters were the same as binary classification, except for the number of top features in each group, we respectively set $d = 80$ and $d = 120$ for Tables 4 and 5. On AD RNA data of prefrontal cortex from MSBB_ArrayTissuePanel, in the bootstrapping stage, we sampled the samples with labels control and AD at a ratio of $17{:}17$. The number of trees was set to 150, the number of initializations for each sampling $p = 100$, the number of sampling $L = 100$, the number of subgroups $q = 5$, and the number of top features in each group $d = 50$. On AD and LATE brain RNA data, for four classes, in the bootstrapping stage, we sampled the samples with LATE+AD, pure LATE, pure AD, and control at a ratio of 41:75:31:90. The number of trees was set to $10,000$, the number of initializations for each sampling $p = 100$, the number of sampling $L = 180$, the number of subgroups $q = 4$, and the number of top features in each group $d = 80$; for pair-wise classes LATE+AD vs. control, pure LATE vs. control, and pure AD vs. control, in the bootstrapping stage, we sampled the samples for these three cases at a ratio of 41:90, 75:90, and 31:90, respectively; for pair-wise classes LATE+AD vs. pure LATE, LATE+AD vs. pure AD, and pure LATE vs. pure AD, we did not perform bootstrapping since their samples were relative balanced; for all these pair-wise classes, the number of sampling $L = 50$ and the number of top features in each group $d = 50$, and the number of trees, the number of initializations, and the number of subgroups were the same as four classes. For all the experiments of these datasets, in the training stage, we randomly divided samples at each RF into training and validation sets at a ratio of 70:30.

In addition, for the hyper-parameters in SVM: in Case 1, for each class we randomly chose 16 as validation samples, the degree of the polynomial kernel was set to four, and all remaining hyper-parameters were set to the default values; in Cases 2 and 3, due to the fewer number of available samples, for each class we randomly chose 11 as validation samples, and the hyper-parameters parameters were set to the default values.

3 Design of synthetic and cross-domain datasets

The following datasets, one is synthetic data with noise background, and one is from the computer vision (CV) field. The second public image datasets is widely used by researchers in the CV field to verify the performance of their proposed algorithms. The dataset is freely accessible; see link at http://yann.lecun.com/exdb/mnist/

#Sample	380	20
Image	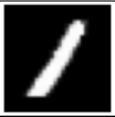	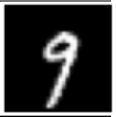
Label	1	9

Table 1: Imbalanced samples 1 vs. 9

#Sample	380	20
Image	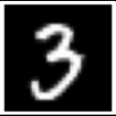	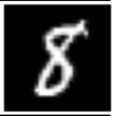
Label	3	8

Table 2: Imbalanced samples 3 vs. 8

#Sample	260	30	70	40
Image	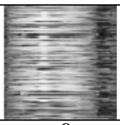	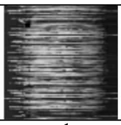	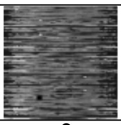	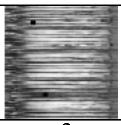
Label	0	1	2	3

Table 3: Four classes of images with or without black points

#Sample	260	30	70	40
Image	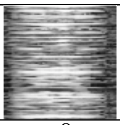	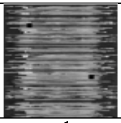	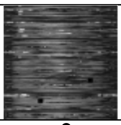	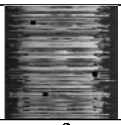
Label	0	1	2	3

Table 4: Four classes of images, with or without cross black points. See Section 3.1 for more details

4 Classification results for synthetic and cross-domain datasets

The classification results and confusion matrix for MNSIT with the labels 1 and 9, and the labels 3 and 8 are shown in the following Tables 5-6 and Figure 1, respectively. The classification results for synthetic data, with or without black points, and the corresponding cross case are shown in the following Tables 7-8 and Figure 2, respectively.

Class	Precision	Recall	F1 score	support
1	0.975	0.995	0.984	27
9	0.979	0.893	0.920	6
Accuracy	\	\	0.975	33
Macro average	0.977	0.944	0.956	33
Weighted average	0.976	0.975	0.974	33

Table 5: Classification results on MINIST with the labels 1 and 9

Class	Precision	Recall	F1 score	support
3	0.949	0.992	0.968	27
8	0.961	0.786	0.847	6
Accuracy	\	\	0.950	33
Macro average	0.955	0.889	0.909	33
Weighted average	0.953	0.950	0.945	33

Table 6: Classification results on MINIST with the labels 3 and 8

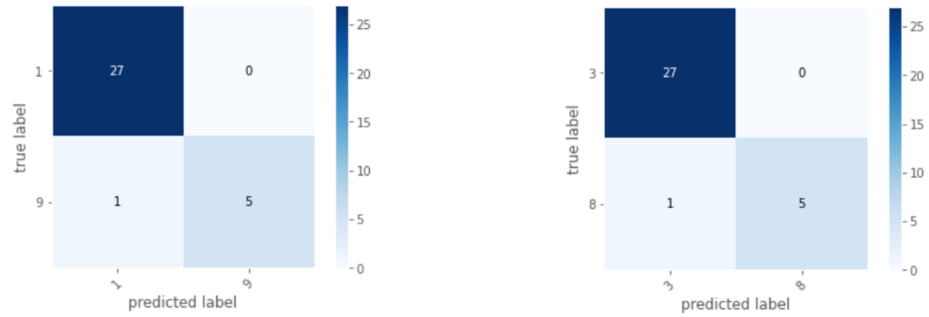

(a) (b)

Figure 1: Confusion matrix for MNIST. (a) For the labels 1 and 9; (b) For the labels 3 and 8

Class (Table 3)	Precision	Recall	F1 score	Support
0	0.665	0.855	0.743	27
1	0.774	0.535	0.611	9
2	0.713	0.696	0.696	21
3	0.870	0.516	0.633	12
Accuracy	\	\	0.703	69
Macro average	0.755	0.650	0.670	69
Weighted average	0.738	0.703	0.694	69
Use classes 1 and 2 in Table 3	Precision	Recall	F1 score	Support
0	0.868	0.814	0.835	21
1	0.828	0.877	0.848	21
Accuracy	\	\	0.844	42
Macro average	0.848	0.846	0.842	42
Weighted average	0.853	0.844	0.843	42

Table 7: Classification results on synthetic data with or without black points

Class (Table 4)	Precision	Recall	F1 score	Support
0	0.687	0.862	0.759	27
1	0.775	0.536	0.612	9
2	0.708	0.721	0.707	21
3	0.865	0.516	0.632	12
Accuracy	\	\	0.713	69
Macro average	0.759	0.659	0.677	69
Weighted average	0.744	0.713	0.704	69
Use classes 1 and 2 in Table 4	Precision	Recall	F1 score	Support
0	0.867	0.811	0.833	21
1	0.826	0.877	0.846	21
Accuracy	\	\	0.842	42
Macro average	0.846	0.844	0.840	42
Weighted average	0.851	0.842	0.841	42

Table 8: Classification results on synthetic data with or without cross black points

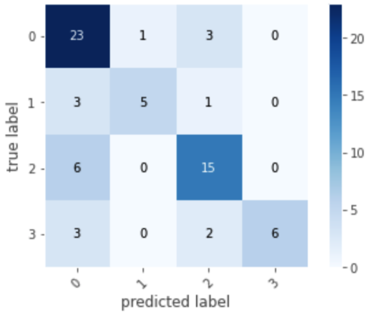

(a)

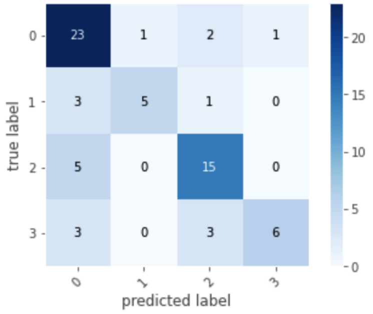

(b)

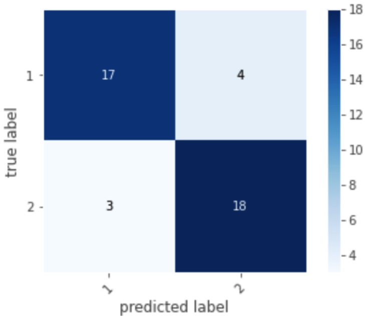

(c)

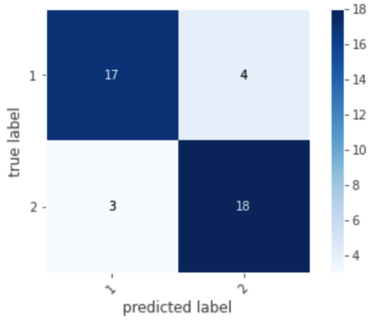

(d)

Figure 2: Confusion matrix for synthetic data. (a) Images with or without black points for all classes in Table 3; (b) Images with or without cross black points for all classes in Table 4. (c) Using classes 1 and 2 in Table 3; (d) Using classes 1 and 2 in Table 4

5 Classification results for RNA expression data

Class	Precision	Recall	F1 score	Support
LATE+AD	0.982	0.504	0.648	11
pure LATE	0.849	0.521	0.636	23
pure AD	1.000	0.487	0.641	10
control	0.561	0.932	0.699	28
Accuracy	\	\	0.674	72
Macro average	0.848	0.611	0.655	72
Weighted average	0.778	0.674	0.663	72

Table 9: Classification results on RNA expression data

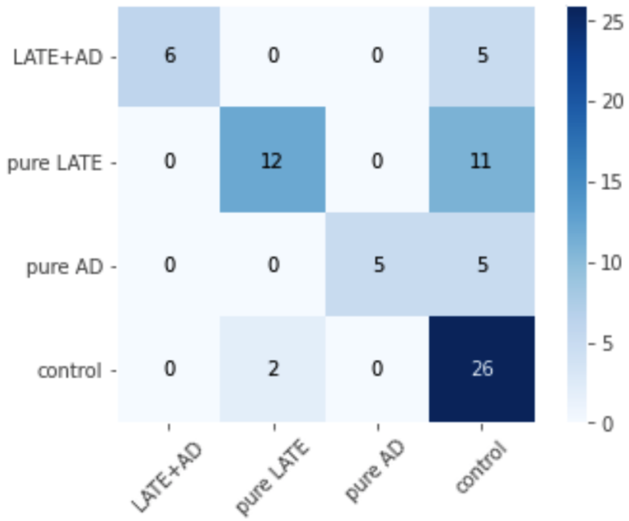

Figure 3: Confusion matrix for RNA expression data. The vertical axis denotes the true labels, and the horizontal axis denotes the predicted labels

6 Comparison of different algorithms

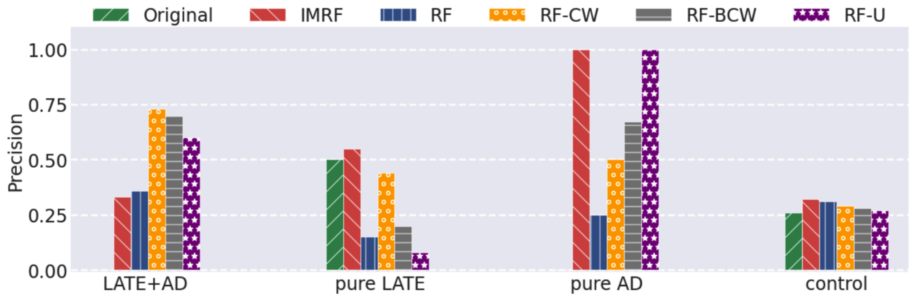

(a)

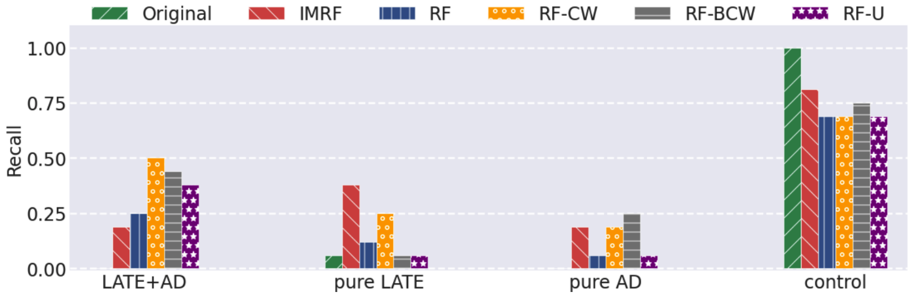

(b)

Figure 4: Comparison of precisions and recalls using the total genes and using the genes selected by different algorithms

7 Identified genes from different algorithms

Algorithm	Gene name (p-value)
RF	*HS.97955* (2.56E-1), ***C2ORF61*** (2.83E-3), *VPS54* (2.92E-1), *LOC651914* (1.41E-1), *KLC4* (5.51E-1), *HS.545258* (3.94E-2), ***HS.542777*** (7.76E-05), *ZBTB7A* (9.07E-3), *HS.545502* (3.48E-1), *ACOX1* (1.83E-05), *HS.568485* (5.01E-2), ***HS.540598*** (1.58E-3), *FLJ45831* (2.61E-1), ***LOC440934*** (2.65E-4), *AGAP3* (2.12E-2), *TUB* (3.45E-1), *NAP1L2* (5.29E-3), *SLC2A9* (3.43E-1), *SNORD64* (1.72E-1), *LRRC36* (1.60E-1), *HS.300217* (1.63E-1), *DEGS1* (9.27E-4), *HS.533995* (7.10E-3), *CHDH* (3.08E-2), *HS.579547* (6.67E-1), *LOC648699* (4.79E-1), *GPR143* (8.29E-1), *CRYGN* (1.20E-1), *SLC43A3* (6.34E-1), ***MED25*** (5.37E-06), *HS.380737* (2.10E-1)
RF-CW	***CLEC7A*** (7.12E-1), ***LOC441546*** (7.00E-4), *NKIRAS2* (8.39E-1), *ING1* (3.35E-1),*HS.568058* (1.87E-2), *LOC643665* (2.18E-1), *HS.156651* (6.42E-4), ***SGCD*** (6.74E-1), *GPC2* (6.72E-3), *ZMAT3* (2.73E-1), *C8ORF58* (4.77E-3), *EPGN* (1.51E-3), *HS.131229* (8.16E-2), *SRY* (4.19E-2), *MCF2L2* (2.09E-2), *LOC646173* (1.02E-1), *NLF2* (8.98E-1), *MAGEB1* (9.91E-1), *VAV3* (1.68E-1), ***STARD7*** (6.84E-05), *CMTM3* (2.42E-1), *HS.574894* (8.00E-2), *ZNF230* (7.45E-1), ***KEAP1*** (1.42E-3), ***SEC31B*** (2.83E-2), *LOC132203* (3.58E-1), *HS.565557* (7.34E-3), *BMP2K* (5.80E-1), *CARD11* (1.62E-2), *WISP3* (9.35E-1), *ALAD* (4.79E-3)
RF-BCW	***CLEC7A*** (7.12E-1), ***LOC441546*** (7.00E-4), *NKIRAS2* (8.39E-1), *ING1* (3.35E-1), *HS.568058* (1.87E-2), *LOC643665* (2.18E-1), *HS.156651* (6.42E-4), ***SGCD*** (6.74E-1), *GPC2* (6.72E-3), *ZMAT3* (2.73E-1), *C8ORF58* (4.77E-3), *HS.131229* (8.16E-2), *EPGN* 1.51E-3), *MCF2L2* (2.09E-2), *SRY* (4.19E-2), *MAGEB1* (9.91E-1), *NLF2* (8.99E-1), *LOC646173* (1.02E-1), ***STARD7*** (6.84E-05), *VAV3* (1.682E-1), *CMTM3* (2.42E-1), *HS.565557* (7.34E-3), *HS.574894* (8.00E-2), *BMP2K* (5.80E-1), *ZNF230* (7.45E-1), ***KEAP1*** (1.42E-3), *ALAD* (4.79E-3), *LOC132203* (3.58E-1), *WISP3* (9.35E-1), ***SEC31B*** (2.83E-2), *HS.544346* (5.77E-2)
RF-U	*NKIRAS2* (8.39E-1), ***CLEC7A*** (7.12E-1), *HS.156651* (6.42E-4), *ING1* (3.35E-1), *LOC643665* (2.18E-1), *EPGN* (1.51E-3), *HS.568058* (1.87E-2), *ZMAT3* (2.73E-1), *LOC646173* (1.02E-1), *GPC2* (6.72E-3), *NLF2* (8.98E-1), ***SGCD*** (6.74E-1), ***LOC441546*** (7.00E-4), *WISP3* (9.35E-1), *MAGEB1* (9.91E-1), *HS.574894* (8.00E-2), *LOC732162* (9.86E-1), *FGF16* (8.30E-3), *LOC132203* (3.58E-1), *LOC126075* (2.21E-1), *ZNF230* (7.45E-1), *SRY* (4.19E-2), *VAV3* (1.68E-1), *BMP2K* (5.80E-1), *HS.560742* (4.57E-3), *MCF2L2* (2.09E-2), *CSDE1* (6.80E-2), *PCDH7* (3.61E-1), ***STARD7*** (6.84E-05), *PAK4* (5.18E-2), *HS.159053* (2.70E-2)

Table 10: Genes identified from 48803 genes by four different kinds of RF-based algorithms. The genes in bold are also selected by our algorithm IMRF, which are shown in Table 2. For RF, there are 18 genes with p-values greater than 0.05; for RF-CW, there are 17 genes with p-values greater than 0.05; for RF-BCW, there are 18 genes with p-values greater than 0.05; for RF-U, there are 20 genes with p-values greater than 0.05

8 Hyper-parameter sensitivity analysis for IMRF

In IMRF, there are mainly 4 more hyper-parameters than traditional RF, that is, the number of initializations for each sampling p, number of sampling L, number of subgroups q, and number of top features in each group d. Next, we will give theoretical and empirical analyses of these new hyper-parameters.

Among these hyper-parameters, the number of initializations per sampling p represents the ergodic number of random states in RF. If p is too small, then the gene subset identified in each sampling would not be stable. However, if p is too large, it would be time consuming. For L, if we use a relatively small one, the perturbation of selected genes is likely to increase. In this paper, we tuned them based on the Results stage in IMRF (i.e., the last stage in Figure 1 in the paper), and we followed the validation results to choose the optimal ones. Therefore, our sensitivity analyses are mainly for the other two hyper-parameters by examining the effects of different q and different d on the performance of IMRF.

Theoretically, if we use smaller d or larger q, we can get the "most" important disease-specific genes; if we use larger d or smaller q, maybe some "less" important disease-specific genes will be included. With consistent IMRF, the "most" important disease-specific genes obtained by smaller d or larger q should be contained in these obtained by larger d or smaller q.

Different numbers of subgroups q

We keep the other hyper-parameter settings of IMRF in the Supplementary Section 2, and we only vary the number of subgroups q to 2, 4, and 6. The calculated results are given in Figure 5. It is seen that, although the number of genes in the identified subsets increases with decreasing q, the resulting gene subsets show a significant subordinate relationship. So, it is evident that IMRF is consistent for different q.

Different numbers of top features in each group d

We keep the other hyper-parameter settings of IMRF in the Supplementary Section 2, and we only vary the number of top features in each group d to 40, 60, and 80. The computed results are provided in Figure 6. It is observed that, although the number of genes in the identified subsets increases with the increasing d, the resulting gene subsets also show a significant subordinate relationship. Thus, it is clear that IMRF is consistent for different d.

Finally, after tuning these hyper-parameters, we picked up 31 genes as the disease-specific gene subset, which is also appropriate from the improved performance of downstream classifier and the superior performance over the other algorithms in comparison.

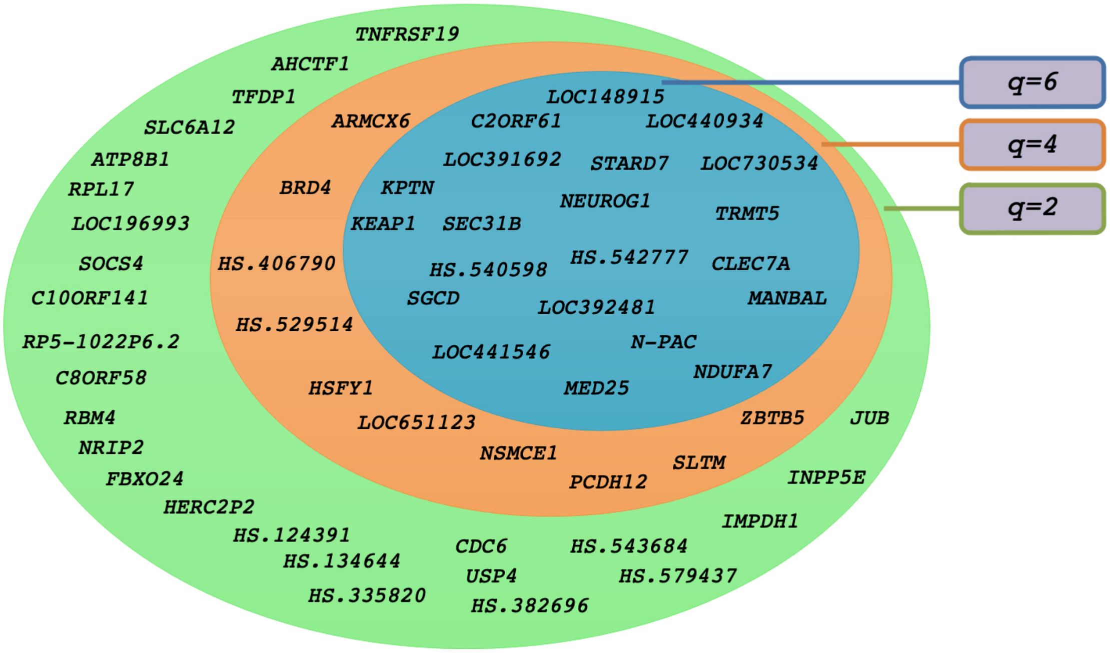

Figure 5: Identified gene subsets for different q values.

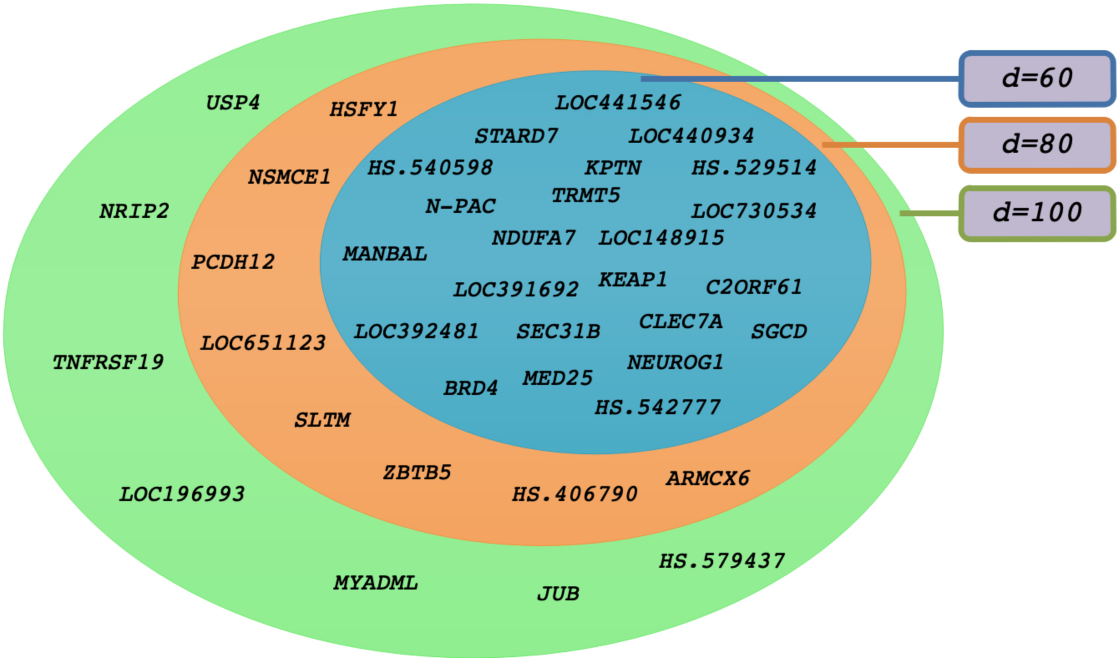

Figure 6: Identified gene subsets for different d values.

9 Codes

The main codes of IMRF can be found at

https://drive.google.com/drive/folders/1XA0fzrJurl1TLkniv9qRdms5-I1QcWN8?usp=sharing

We will make all the codes publicly available on GitHub upon acceptance of our paper.
